# Community engagement strategies for genomic studies in Africa: a review of the literature

**DOI:** 10.1186/s12910-015-0014-z

**Published:** 2015-04-12

**Authors:** Paulina Tindana, Jantina de Vries, Megan Campbell, Katherine Littler, Janet Seeley, Patricia Marshall, Jennifer Troyer, Morisola Ogundipe, Vincent Pius Alibu, Aminu Yakubu, Michael Parker, as members of the H3A Working Group on Ethics

**Affiliations:** Navrongo Health Research Centre, Ghana Health Service, P.O. Box 114, Navrongo, Ghana; Department of Medicine, University of Cape Town, Office J52-16, UCT Centre for Clinical Research, Old Main Building, Groote Schuur Hospital, Observatory, 7925 Cape Town, South Africa; Department of Psychiatry and Mental Health, University of Cape Town, J-Block, Groote Schuur, Observatory, 7925 Cape Town, South Africa; Wellcome Trust, Gibbs Building, 215 Euston Road, London, NW1 2BE UK; MRC/UVRI Uganda Research Unit on AIDS, P.O. Box 49, Entebbe, Uganda; Department of Bioethics, School of Medicine, Case Western Reserve University, 10900 Euclid Avenue, Cleveland, Ohio 44106-4976 USA; National Human Genome Research Institute, National Institute of Health, Bethesda, USA; West African Bioethics Training Program, Center for Bioethics, 102 Bashorun Road, Ibadan, Oyo State; College of Natural Sciences, Department of Biochemistry and Sports Science, Makerere University, P.O. BOX 7062, Kampala, Uganda; National Health Research Ethics Committee, Federal Ministry of Health, Abuja, Nigeria; The Ethox Centre, University of Oxford, Oxford, UK

**Keywords:** Community engagement, Genetics, Genomic research, Africa

## Abstract

**Background:**

Community engagement has been recognised as an important aspect of the ethical conduct of biomedical research, especially when research is focused on ethnically or culturally distinct populations. While this is a generally accepted tenet of biomedical research, it is unclear what components are necessary for effective community engagement, particularly in the context of genomic research in Africa.

**Methods:**

We conducted a review of the published literature to identify the community engagement strategies that can support the successful implementation of genomic studies in Africa. Our search strategy involved using online databases, Pubmed (National Library of Medicine), Medline and Google scholar. Search terms included a combination of the following: community engagement, community advisory boards, community consultation, community participation, effectiveness, genetic and genomic research, Africa, developing countries.

**Results:**

A total of 44 articles and 1 thesis were retrieved of which 38 met the selection criteria. Of these, 21 were primary studies on community engagement, while the rest were secondary reports on community engagement efforts in biomedical research studies. 34 related to biomedical research generally, while 4 were specific to genetic and genomic research in Africa.

**Conclusion:**

We concluded that there were several community engagement strategies that could support genomic studies in Africa. While many of the strategies could support the early stages of a research project such as the recruitment of research participants, further research is needed to identify effective strategies to engage research participants and their communities beyond the participant recruitment stage. Research is also needed to address how the views of local communities should be incorporated into future uses of human biological samples. Finally, studies evaluating the impact of CE on genetic research are lacking. Systematic evaluation of CE strategies is essential to determine the most effective models of CE for genetic and genomic research conducted in African settings.

## Background

Community engagement (CE) has been broadly defined as a process of working collaboratively with a group or groups of people on a shared goal or common interest [1]. It has been recognised as an important activity that can promote the ethical conduct and successful implementation of research by ensuring that research is locally relevant to the host community and that local perspectives are incorporated into the design and conduct of research [2,3]. It is also important in extending the ethical principle of ‘respect for persons’ to entire communities, avoiding exploitation, and building trust between researchers and the communities involved in research [4,5]. For example the HIV Prevention Trials Network (HPTN) has suggested that research projects on socially sensitive issues are most likely to succeed if communities are actively involved in the research process, from inception through to the dissemination of findings [6].

With the advent of genetic and genomic research, there have been calls for the development of genuine partnerships with relevant populations as well as communities encompassing the sample donors [7,8]. Genetic research differs from other biomedical research in that genetic background, while contributing to disease state, is not a temporary condition or a unique attribute of those with the disease, but rather an enduring set of information that is shared with family members, relatives, and even entire populations and communities to differing degrees. In addition, genetic background can be stigmatizing. Genetic predisposition to certain diseases or membership in certain racial or ethnic groups can have negative social, political, and economic consequences in some contexts. Finally, genetic and genomic concepts may not be well understood, especially in under-resourced or isolated communities, including health professionals [8-12]. Not coincidentally, these communities are often the subjects of the most productive genetic research exactly because of their isolation and the relative homogeneity of their genomic context. At the same time, the potential benefits to these communities, while long-term, can be quite large if effective CE strategies are implemented.

CE in the context of genomic research provides opportunities for informing and educating communities about genomics and genomic research, and exchanging information between the research team and potential research participants about the research process over a period of time. In some research settings, CE may be conducted prior to data collection. In other settings, CE may be continued throughout the duration of a study. CE may enhance understanding of research goals and procedures particularly with the complexities involved in genomic studies. It also provides an avenue for feeding back research findings to participants and communities [10,11]. Therefore, ideally, CE activities should occur prior to, during and after a research project.

Despite general agreement on the intrinsic and instrumental values of CE and the growing scholarship on the practice of CE in global health research [1,13-19], questions remain about the potential impact of various CE methods, approaches and models and it is not clear what constitutes effective community engagement, particularly in the context of genomic research in Africa.

The Human Heredity and Health in Africa initiative (H3Africa) is an international collaborative project that aims to build genomic research capacity in Africa [20]. Many of the H3Africa projects involve the collection of human biological samples from research participants in African populations. One of the key ethical issues identified by the H3Africa Working Group on Ethics is community engagement. Given the lack of knowledge and limited guidance for the actual practice of CE, the Working Group set out to identify examples of effective CE strategies and models that can support the successful implementation of H3Africa projects. The goal is for recommendations drawn from this study to inform the Community Engagement guidelines for the H3Africa consortium and the engagement strategies that will be carried out by H3Africa research projects as well as other projects undertaking genomic studies in Africa in the future.

## Methods

A review of literature on community engagement in the context of biomedical and genomic research in Africa was conducted between October 2013 – May 2014 using Pubmed (National Library of Medicine), Medline and Google scholar. Key words included a combination of the following: community engagement, community advisory boards, community consultation, community participation, effectiveness, genetic and genomic research, Africa, developing countries. The authors’ own experiences and prior knowledge of research groups in Africa conducting similar work on community engagement further facilitated the identification and selection of research articles.

### Selection criteria for articles

Published articles and reports on community engagement/community involvement/community advisory boards in biomedical research and genetic and genomic research were selected. Given the focus of this study on genomics, primary studies of CE or ‘actual experiences’ of engaging with communities in specific research contexts in Africa published between 2003 (when the Human Genome Project was completed) and 2014 were prioritized,. Commentaries or opinion pieces on community engagement, as well as articles unrelated to (bio) medical research (including genetic and genomic research) in Africa were excluded. Thus, although there is a wealth of information on community engagement in the context of health promotion, health interventions and community development, these reports were not considered relevant for this analysis and were excluded. Only articles in English with full text available online were considered. The concept and practice of *public engagement* will be discussed in a separate manuscript to address issues specific to biobanks in the context of H3Africa.

### Data extraction

The process of data extraction involved a critical review of the selected articles to identify the following information: type of primary study; definition of community; target community for CE; definition of CE; CE methods, model or strategy; CE rationale; underlying principles; methods used for evaluation; and lessons learned. Particular attention was given to primary studies on community engagement for biomedical, genetic and genomic research; methods used in these studies; whether the studies included an evaluation of the CE strategy/approaches/methods; and how effectiveness and success of a given CE strategy were measured.

## Results

**Figure 1:**
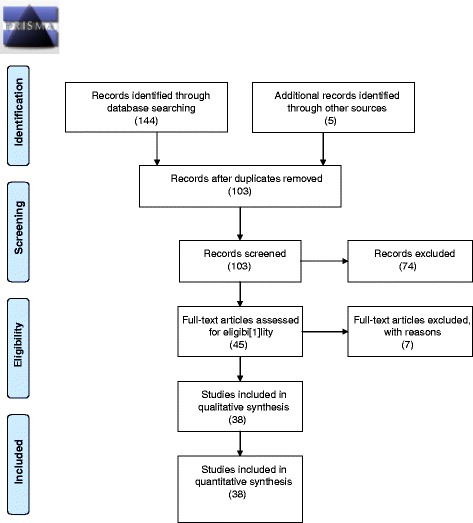
PRISMA flow diagram.

In total 44 articles and 1 MSc thesis [21] were retrieved based on the selection criteria described above. After a careful review of the abstracts of these articles, 38 articles specific to community engagement in sub-Saharan Africa were selected (see Figure 1). Of these, 21 were primary studies on community engagement while the rest were reports of ‘experiences’ or ‘lessons’ extracted from engaging with communities in the context of biomedical research projects. Of the 38 articles included in this review 34 related to biomedical research (including clinical trials) generally, while 4 were specific to genetic and genomic research in Africa [10,11,18,19]. A total of 8 articles focused specifically on the role and functions of community advisory boards (CABS) [22-29]. Tables 1 and 2 present a bibliography of the key articles included in this review.

**Table 1:** **Selected articles on community engagement in biomedical research in Africa**

1	Bandewar, S.V., Kimani, J and Lavery, J.V. 2010. The origins of a research community in the Majengo Observational Cohort Study, Nairobi, Kenya. BMC Public Health, 10,630. **(Kenya)** [35]
2	Boga M, Davies A, Kamuya D, Kinyanjui SM, Kivaya E, Kombe F, Lang T, Marsh V, Mbete B, Mlamba A *et al.*: Strengthening the informed consent process in international health research through community engagement: the KEMRI-Wellcome Trust Research Programme Experience. PLoS Med. 2011;8(9):e1001089. **(Kenya)** [44]
3	Chantler T, Otewa F, Onyango P, Okoth B, Odhiambo F, Parker M, Geissler PW. Ethical challenges that arise at the community interface of health research: village reporters’ experiences in Western Kenya. Dev World Bioeth. 2013 Apr;13(1):30-7. **(Kenya)** [17]
4.	Cohen E. Community Engagement in Globl Health Research: Case studies from the developing world: The Zomba District, Malawi Case Study. Masters’ Thesis 2008. Simon Fraser University, Canada. [21]
5.	The HapMap Consortium (2004). “Integrating science and ethics in the International HapMap Project.” Nature Reviews Genetics 5: 467-675. (global including Nigeria) [8]
6.	Cox LE, Rouff JR, Svendsen KH, Markowitz M, Abrams DI. Community advisory boards: their role in AIDS clinical trials. Terry Beirn Community Programs for Clinical Research on AIDS. Health Soc Work. 1998;23(4):290–7. [25]
7.	Fairhead, J., M. Leach, and M.. Public engagement with science? Local understandings of a vaccine trial in the Gambia. J Biosoc Sci. 2006;38(1):103–16. **(Gambia)**
8.	Family Health International. Community involvement in international research: lessons learned from the HIV Prevention Trials Network. Research Triangle Park, NC: Family Health International, 2006. **(International including Tanzania, Uganda, Zimbabwe, Zambia)** [6]
9.	Grinker RR, Chambers N, Njongwe N, Lagman AE, Guthrie W, Stronach S, et al. “Communities” in community engagement: lessons learned from autism research in South Korea and South Africa. Autism Res. 2012 Jun; 5(3):201-10. doi:10.1002/aur.1229. Epub 2012 May 4. (South Africa) [32]
10.	Kamanda A, Embleton L, Ayuku D, Atwoli L, Gisore P, Ayaya S, Vreeman R, Braitstein P. Harnessing the power of the grassroots to conduct public health research in sub-Saharan Africa: a case study from western Kenya in the adaptation of community-based participatory research (CBPR) approaches. BMC Public Health. 2013;13:91.
11.	Kamuya, D. M., V. Marsh, et al. (2013). “Engaging communities to strengthen research ethics in low-income settings: selection and perceptions of members of a network of representatives in coastal kenya.” Developing world bioethics 13(1): 10-20. (Kenya) [45]
12.	Koen J, Essack Z, Slack C, Lindegger G, Newman PA. ‘It looks like you just want them when things get rough’: civil society perspectives on negative trial results and stakeholder engagement in HIV prevention trials. Dev World Bioeth. 2013 Dec;13(3):138-48. **(South Afria)** [52]
13.	Lang T, Gould J, Seidlein Von L, Lusingu JP, Mshamu S, Ismael S, Liheluka E, Kamuya D, Mwachiro D, Olotu A, Njuguna P, Bejon P, Marsh V, Molyneux C: Approaching the community about screening children for a multicentre malaria vaccine trial. Int Health 2012, 4:47–54. **(Kenya)** [48]
14.	Marsh V, Kamuya D, Rowa Y, Gikonyo C, Molyneux S: Beginning community engagement at a busy biomedical research programme: experiences from the KEMRI CGMRC-Wellcome Trust Research Programme, Kilifi, Kenya. Soc Sci Med. 2008;67(5):721–33. (Kenya) [16]
15.	Magnus M, Franks J, Griffith S, Arnold MP, Goodman K, Wheeler DP; for the HPTN 061 Study Group. Engaging, Recruiting, and Retaining Black Men Who Have Sex With Men in Research Studies: Don’t Underestimate the Importance of Staffing-Lessons Learned From HPTN 061, the BROTHERS Study. J Public Health Manag Pract. 2014 Jan 9. (South Africa) [36]
16.	Mitchell K, Nakamanya S, Kamali A, Whitworth JA. “Exploring the community response to a randomized controlled HIV/AIDS intervention trial in rural Uganda.” AIDS Educ Prev. 2002;14(3):207–16
17.	Morin, S. F., A. Maiorana, et al. (2003). “Community consultation in HIV prevention research: a study of community advisory boards at 6 research sites.” J Acquir Immune Defic Syndr **33**(4): 513-520. **(South Africa)** [23]
18.	Morin, S. F., S. Morfit, et al. (2008). “Building community partnerships: case studies of Community Advisory Boards at research sites in Peru, Zimbabwe, and Thailand.” Clin Trials **5**(2): 147-156. **(Zimbabwe)** [22]
19	Mosavel, M., C. Simon, et al. (2005). “Community-based participatory research (CBPR) in South Africa: engaging multiple constituents to shape the research question.” Soc Sci Med **61**(12): 2577-2587. **(South Africa)** [41]
19.	Nakibinge, S., D. Maher, et al. (2009). “Community engagement in health research: two decades of experience from a research project on HIV in rural Uganda.” Trop Med Int Health 14(2): 190-195. **(Uganda)** [37]
20.	Nyika A, Chilengi R, Ishengoma D, Mtenga S, Thera MA, Sissoko MS, Lusingu J, Tiono AB, Doumbo O, Sirima SB *et al.*. Engaging diverse communities participating in clinical trials: case examples from across Africa. Malar J. 2010;9:86. (Mal, Tanzania) [43]
21.	Ntshanga SP, Ngcobo PS, Mabaso ML. Establishment of a Community Advisory Board (CAB) for tuberculosis control and research in the Inanda, Ntuzuma and KwaMashu (INK) area of KwaZulu-Natal, South Africa. Health Policy. 2010;95(2-3):211–5. South Africa. [28]
22.	Okello G, Jones C, Bonareri M, Ndegwa SN, McHaro C, Kengo J, Kinyua K, Dubeck MM, Halliday KE, Jukes MC, Molyneux S, Brooker SJ. Challenges for consent and community engagement in the conduct of cluster randomized trial among school children in low income settings: experiences from Kenya. Trials. 2013 May 16;14:142. (Kenya) [23]
23.	Participants in the Community Engagement and Consent Workshop, Kilifi, Kenya, March 2011 Consent and Community Engagement in diverse research contexts Author(s): Source: Journal of Empirical Research on Human Research Ethics: An International Journal, Vol. 8, No. 4 (October 2013), pp. 1-18 **(Global including Africa)** [15]
24.	Reddy P, Buchanan D, Sifunda S, Shamagonam J, Naidoo N. The role of community advisory boards in health research: Divergent views in the South African experience. SAHARA J. 2010;7(3):2–8. **South Africa**. [24]
25.	Seeley, J.A., Kengeya-Kayondo, J.F., and Mulder, D.W. (1992). Community-based HIV/AIDS research — whither community participation? Unsolved problems in a research programme in rural Uganda. Social Science and Medicine 34(10):1089-95. **(Uganda)** [34]
26.	Simon Christian, Mosavelb Maghboeba, Stade van Debbie. Ethical challenges in the design and conduct of locally relevant international health research. Social science &Medicine. Vol 64, Issue 9, may 2007, Pg 1960-1969. **(South Africa)** [29]
27.	Stadler J, Dugmore C, Venables E, MacPhail C, Delany-Moretlwe S. Cognitive mapping: using local knowledge for planning health research. BMC Med Res Methodol. 2013;13:96. **South Africa**. [51]
28.	Strauss, R. P., S. Sengupta, et al. (2001). “The role of community advisory boards: involving communities in the informed consent process.” American journal of public health 91(12): 1938-1943. **(South Africa)** [26]
29.	Shagi C, Vallely A, Kasindi S, Chiduo B, Desmond N, Soteli S, et al. A model for community representation and participation in HIV prevention trials among women who engage in transactional sex in Africa. AIDS Care. 2008;20(9):1039–49. **South Africa**. [30]
30.	Shubis, K., O. Juma, et al. (2009). “Challenges of establishing a Community Advisory Board (CAB) in a low-income, low-resource setting: experiences from Bagamoyo, Tanzania.” Health Res Policy Syst 7: 16. **(Tanzania)** [27]
31.	Strauss, R. P., S. Sengupta, et al. (2001). “The role of community advisory boards: involving communities in the informed consent process.” American journal of public health 91(12): 1938-1943. **(South Africa)**
32.	Tedrow VA, Zelaya CE, Kennedy CE, Morin SF, Khumalo-Sakutukwa G, Sweat MD, Celentano DD. No “magic bullet”: exploring community mobilization strategies used in a multi-site community based randomized controlled trial: Project Accept (HPTN 043). AIDS Behav. 2012 Jul;16(5):1217-26. **(Multi site including South Africa)** [39]
33.	Tekola, F., Bull, S. J., Farsides, B., Newport, M. J., Adeyemo, A., Rotimi, C. N., & Davey, G. (2009b). Tailoring consent to context: Designing an appropriate consent process for a biomed- ical study in a low-income setting. PLoS Neglected Tropical Diseases, 3(7), e482. **(Ethiopia)**
34.	Tindana, P. O., L. Rozmovits, et al. (2011). “Aligning community engagement with traditional authority structures in global health research: a case study from northern Ghana.” American journal of public health **101**(10): 1857-1867. **(Ghana)** [5]

**Table 2:** **Primary studies specific to Community engagement in genetic and genomic research in Africa**

1	Marsh VM, Kamuya DM, Mlamba AM, Williams TN, Molyneux SS. Experiences with community engagement and informed consent in a genetic cohort study of severe childhood diseases in Kenya. BMC Med Ethics. 2010;11:13. **(Kenya)** [18]
2	Marsh VM, Kombe F., Fitzpatrick R, Williams TN, Parker M, Molyneux CS. Consulting communities on feedback of genetic findings in international health research: sharing sickle cell disease and carrier information in coastal Kenya. BMC Medical Ethics 10/2013; 14(1):41. **(Kenya)** [19]
3	Rotimi, C., M. Leppert, et al. (2007). “Community engagement and informed consent in the International HapMap project.” Community genetics **10**(3): 186-198. **(International including Nigeria)** [11]
4	Tindana, P., S. Bull, et al. (2012). “Seeking consent to genetic and genomic research in a rural Ghanaian setting: A qualitative study of the MalariaGEN experience.” BMC medical ethics **13**(1): 15. **(Ghana)** [10]

### How is the concept of community defined?

One of the challenges with identifying an effective CE strategy for any research project is the lack of uniformity in the way concepts such as ‘community’ and ‘community engagement’ are defined. There is broad agreement in the literature that the definition of community largely depends on the nature of the proposed research, the goal of engagement and the context in which the research is carried out. In this review, community in biomedical research was broadly defined in relation to the geographic location of the research project and the target group to be engaged, particularly disease specific projects [5,11,30-33]. Seeley et al. defined community as the ‘population under study’ – defined by a geographical area [34]. These authors acknowledged that there are several communities in the area and not everyone participates in the research. To Morin et al., community is more than the target population of the research and includes people affected by the research and vulnerable populations more generally [22,23]. These groups might not necessarily live within the same geographic area. Bandewar et al. identified their community in relation to a common trade shared by its members (women who engaged in sex work) and by locality (Nairobi in Kenya) [35].

In the context of genomic research, Rotimi et al. used the terms ‘populations’ and ‘communities’ in the HapMap project. They defined population as a group of people who have a common geographic ancestry and the term community as ‘a group of individuals within the population who shared common characteristics of social integration’ [11]. Marsh et al. conceptualised communities in relation to their project’s geographic location namely the Kilifi district, identifying groups within the district affected by sickle cell disease [19]. Tindana et al. defined community in relation to common ethnicities, languages and location, resulting in two communities being identified for engagement namely the Kassena and Nankana communities of northern Ghana [10].

There was no uniformity in how community was defined in the articles reviewed. In determining the target ‘community’ to be engaged, most researchers focused on specific geographic locations from which potential research participants or sample donors would be recruited, the common characteristics (ethnicity [10], disease [31-34], trade [30,35], sexual orientation [36]) shared by members of a group or groups within these locations and those that were likely to be directly affected by the research [37,38]. It is interesting to note that in all cases the community was defined by the research team. While this is typical of community engagement processes within biomedical research, it does impact on the type of representation achieved with these engagement processes.

### Clarifying the concepts: what is community engagement?

Most of the articles reviewed did not specifically provide a definition of CE but made reference to project goals and rationales for engaging the targeted communities, as well as the strategies used in the engagement process. For those that did, CE was defined broadly as a process of working collaboratively with a group or groups of people on a shared goal or common interest [1,37]. Other related concepts such as community involvement, community participation, community consultation and community collaboration were used interchangeably with CE depending on the level of engagement [5,34,35,39]. Some articles used the term Community-Based Participatory Research (CBPR), drawing from participatory action research, which is generally defined as a collaborative approach to research with equitable involvement of partners in *all* aspects of the research [40-42]. CBPR uses specific methodologies to engage the target community including identifying a specific research problem, planning the research and disseminating the findings [40]. This approach to research was particularly common in HIV/AIDS research. Morin et al. have suggested that a Community Advisory Board (CAB) is one approach to CBPR, which aims to strengthen partnerships with communities in socially sensitive research contexts such as HIV/AIDS research [22]. According to Cox et al., CABs have been defined as ‘active, duly organised and representative bodies that hold regular meetings and make decisions on behalf of their members’ [25].

In the context of genomic research, Marsh et al. defined CE within the framework of action research, which aimed to bring together action and reflection, in collaboration with researchers and the community. The goal was to arrive at practical solutions to problems raised within the research process, specifically how findings of a sickle cell study ought to be fed back to research participants [19]. On the other hand, Rotimi et al. explained that their engagement process was not aimed at arriving at a consensus. Rather, the goal was to provide members of the communities with information about the project, and in turn give them the opportunity to share their views about the ethical, social and cultural issues the project raised for them, their immediate communities, and the broader communities and populations of which they were a part [11]. In our review, we draw on the typology developed by the International Association of Public participation (IAP2) spectrum –*inform/consult/involve/collaborate/empower (**www.iap2.org**)*, and what Seeley et al. also refer to as the *contract/consultative/collaborative/collegiate* framework to identify the level of engagement involved in these articles [34].

#### Information giving/sharing

Almost all the articles mentioned ‘*providing or sharing information’* as a necessary requirement in the engagement process prior to the initiation of a research project. This is not surprising considering that genomic research is often complex, and many African communities are unfamiliar with the research process generally, and genomics research more specifically. This unfamiliarity is further compounded by language and cultural barriers between the research team and the community, poor education amongst some communities and low levels of literacy [10,13]. As a result, information giving and sharing becomes an important way of building rapport and empowering the community.

The aim of providing information is to enhance research understanding and also to support the informed consent process. For some projects, this involved developing educational materials on key aspects of the research project to be shared with the target communities [21]. However, information provision in itself is not enough for a community to be actively engaged in research. In the context of genomic research, Rotimi et al. reported that their engagement strategy aimed to: 1) provide an opportunity for a broad range of members of the target community to contribute opinions about how the samples from their locality would be collected and described; 2) provide extensive information about the project so that decisions about donating samples were better informed; and 3) keep the community informed about how the samples were being used and about findings from future studies based on the HapMap project or the samples [11]. For current genomic studies, information-giving may also be very important at the end of the project to provide feedback on research findings to the community. Some of the articles highlighted the importance of utilising pre-existing CE forums to disseminate research findings to the community [5,11,38].

#### Consultation

Community consultation refers to obtaining [community] feedback on various aspects of a research project. The aim is to ensure that the interests of the community are taken into consideration in the design and conduct of the research. Several articles in this review mentioned consultation with relevant groups within the community such as community leaders, community advisory boards, research ethics committees and community representatives, before approaching individuals for informed consent. These consultations enable the research team to secure buy-in and general support from various stakeholders [39]. Marsh et al. noted that consultation requires ‘authentic community representation, where authenticity implies fair, balanced and accurate representation of the many and varied constituencies within the community’ [16]. Such representation could be from political leaders, religious leaders, community leaders, community advisory boards, or respected individuals within the community. For example, Rotimi et al., Marsh et al., Morin et al. and Tindana et al. all reported that they consulted with and sought the permission of local community leaders before approaching members of the community [10,11,18,22]. Tindana et al. explained that consultation with community leaders was not only a traditional requirement for seeking permission to approach members of the community but also an opportunity to gain insights into cultural values that may have implications for the research project [5]. This seems to be a unique feature of community engagement in rural African settings where the community structure is well defined and recognised and where there is some community cohesiveness [1]. Tedrow et al. also suggested that consultations with community leaders are relatively unproblematic as long as proper administrative procedures are followed [39]. Other authors also noted that community representatives and CABs can also serve as brokers in bringing researchers and the local community together [22]. Consultations can also be extended to the broader community through community meetings as described in the articles by Tindana et al. and Marsh et al. [5,13,16]. However, it was not clear in these papers how feedback from such consultations actually feeds into the design and conduct of other types of research such as genomic studies.

#### Community involvement and collaboration

According to the IAP2 typology we used to guide our analysis, community involvement is a process of working directly with the community or through their representatives to shape the design and conduct of the research project. Involvement goes beyond information giving and consultation. In the context of CBPR, Mosaveli et al. reported that extensive consultations, interviews and focus group discussions with various constituents within the community helped to refocus their research question [41]. Involvement evolves overtime and often requires efforts to sustain the interest of the community in the research process. We were unable to determine from many projects reported in these articles how communities were *involved* in shaping the design of the research project and whether this is appropriate for genomic studies. However, several articles highlighted the CAB model as one of the common and effective approaches to community involvement in research, particularly in HIV/AIDs research and the HapMap Project.

Collaboration involves ‘partnering with the [community] on each aspect of the research including the development of alternatives and the identification of preferred solutions’ [IAP2]. Here, communities or their representatives work closely with the research team to formulate the research questions, decide on the design of the study and be involved in the dissemination of findings. There is a thin line between involvement and collaboration and thus in many of the articles we reviewed, the two were used interchangeably. However, the emphasis on the collaborative nature of the research was more pronounced in articles focusing on the role and functions of CABs and where CE was viewed as an opportunity to build authentic and mutually respectful relationships between the research team and the target community.

Seeley et al. have concluded that the majority of ‘engagement’ in research is at the level of a contract rather than working collaboratively with community members in the design and implementation of the research [34]. This was the case for many of the articles reviewed where the engagement process is based on a research project that has already been designed and in some cases approved by a research ethics committee. Where this is the case, CE may provide an opportunity for the community to identify research needs. For instance, in the work reported by Seeley et al., in a project initially focused on HIV, the community raised concerns about the burden of malaria during the engagement process, which then formed the topic of further studies. Rotimi et al. also reported that some practical aspects of the recruitment and sample collection process for the HapMap project were modified in response to information collected during the community discussions [11]. This has implications for current genomic studies where the aims, objectives and design of the study have already been defined by the research team. However, there is still room for research teams to request for amendments to approved research protocols from research ethics committees or institutional review boards (IRBs) based on the feedback from the community. CE could also provide an opportunity to gauge community views on what future studies could be conducted on the samples that have been collected and stored.

### What methods/models/approaches to community engagement exist for genomic research in Africa?

Various methods and approaches of engaging communities in research exist in the literature. The methods used often depend on the goals of the engagement, the context in which the research is carried out, and the level of engagement. This can differ across sites contributing to the same project. For instance, in the case of HapMap [11], the Malaria Vaccine trials reported by Nyika et al. [43] and Morin et al.’s case studies on CABs in the HIV research [22], engagement strategies differed from site to site because of differences in community structure and cultural norms. Given that the process of informing potential participants and their communities about a proposed research project is key to the engagement process, most of the articles we reviewed used methods and approaches that maximised opportunities for some interaction and exchange of information between the research team and the target community or population. We have grouped the various CE methods/approaches into two categories: Those that involve direct engagement with potential research participants and their communities and those that involve engagement through their representatives. We discuss each of these categories in the following section of the paper.

### Strategies for direct engagement with potential research participants and their communities

#### Town hall meetings/Community gatherings/meetings/forums

Engaging directly with the target ‘community’ often involves approaches that promote direct, face-to-face interactions between the research team and the target community. Examples include town hall meetings, community meetings and public meetings. The aim is often to reach people who are affected by or involved in the proposed research. These approaches are very common in community-based or population-based studies where the views of a wider audience are solicited [5,10,18,44,45]. In some African settings such as Ghana, this kind of community gathering or meeting is referred to as a ‘durbar’ and in others, particularly in East Africa; they are called ‘barazas’ (a Swahili word which refers to a deliberative meeting of people) [5,16]. In the HapMap project, Rotimi et al. reported that they utilised town hall meetings and public forums to disseminate information about the project [11]. These meetings are often attended by various groups within the target community or population such as community leaders, chiefs, opinion leaders and the members of the community. According to Marsh et al., the numbers could range from 50 to 300 people per meeting depending on the research setting [18].

One advantage of this direct method of engagement is that it provides an opportunity for many people affected by or with a stake in the research to have a fair chance of getting first-hand information directly from the research team. These community gatherings also provide an important avenue to solicit the views of a wider audience on various aspects of a proposed project. For a malaria genomic research case study conducted in rural northern Ghana, Tindana et al. reported that the community durbar provided a unique opportunity for the research team to rehearse effective provision of complex information on genomic research which later informed the individual consent process [10]. Marsh et al. have also suggested that community meetings provide an opportunity to open a dialogue between the research team and the community members [16].

Challenges associated with engaging with large groups of people include difficulties in identifying the relevant communities to be engaged in advance of the research [10], difficulties in assessing people’s understanding of the information given in such large meetings and the tendency for the discussions to be dominated by more vocal people in the audience. These strategies may also fail to capture the perspectives of other relevant members of the community who are unable to attend the meeting.

#### Focus group discussions

Another CE method mentioned in our review was the use of small group or focus group discussions. In contrast to community meetings discussed above, these involve fewer people, are more focused and target specific groups of people within the larger community [29]. Examples include meetings with women groups as reported by Tindana et al. [10], meetings with chiefs and opinion leaders as reported in Marsh et al. [18], community development officers, community activists [29] and meetings with patient groups for disease specific projects.

### Engagement through representatives

#### CABs

The CAB model was the most cited example of a community engagement strategy in our review [6,22-29]. According to Shubis et al., CABs have traditionally been defined as “active, duly organized and representative bodies that hold regular meetings and make decisions on behalf of their membership and whose members serve without pay” [27]. Thus, CAB members serve as a liaison between the research teams and research participants. They are expected to bring in the voice of the target community and also feedback information about the research to the community. Morin et al. distinguish between two CAB models, the ‘broad community’ model and the ‘population specific model. The ‘broad community’ model encompasses a wide range of stakeholders including government officials and religious leaders. This model is reported to be particularly useful for an institution-wide CE strategy where the research institution maintains a long-term relationship with the community. The ‘population specific’ model often targets a specific group such as a patient group, with representation limited to a group within the larger community [22]. In some cases, CABs could also be study specific focusing only on one study, or area specific which focuses on several studies within a specific research community.

Many of the articles in this review highlighted the importance of the CAB as a model that can strengthen partnerships between the research team and the target community over a long period of time. However, despite this advantage, the CAB model may not necessarily be appropriate for all types of research. Morin et al. reported that identifying the appropriate people with the time to commit to the project was one of the key challenges of the CAB model [23]. Other challenges include deciding on membership, for example who are the best people to serve on CABs, how should they be selected and what exactly should their role be? If a CE approach is targeting patient groups and associations, then it might be possible for the group to nominate representatives to speak on their behalf. However, for a wider community or population, this could still present several challenges including choosing a representative that would be accepted by the local community. Shubis et al. reported that working in poor communities can also present unique challenges with ‘monetary expectation of CAB representatives’ [27]. These expectations need to be addressed upfront with effective communication between the research team and the CABs.

In many of the articles included in this review the research team appeared to play an active role in a researcher-led community engagement process [10,43-45]. In genomic studies, engaging with communities unfamiliar with the research process also creates a dilemma for the research team. On one hand the research team seeks to engage with the community about how best to manage the research process. One function of the CE activity may be to hold the research team accountable for best practice. But on the other hand the research team has to inform, educate and empower that same community about genomic research first in order for them to play this role. This process takes time. As a result, while there is a degree of bias inherent in the research team being involved in the community engagement activities, it may also be a very necessary and appropriate step in building community engagement within these communities.

### What methods are used to assess the effectiveness of CE?

One of the objectives of this project was to identify *effective* community engagement strategies that can support genomic studies in Africa. Most of the articles reviewed did not systematically evaluate their community engagement approach or method. Marsh et al. reported that given the complexities in the goals and mechanisms used for CE, determining the effectiveness of a CE strategy remains a challenge [16]. However several articles, particularly the primary studies we identified, used case studies within participatory action research, and qualitative research methods involving ethnographic fieldwork, in-depth interviews and focus group discussions to elicit the views of community members on the engagement strategy used in the projects [17,35,40-47]. While this feedback was not evaluative, it did provide helpful insights into community members’ experiences of community engagement processes. Tedrow et al. used a qualitative multi-case design [39] while Tindana et al. used focus group discussions and in-depth interviews with specific groups in the community to consider how CE enhanced research understanding and the informed consent process [10]. Marsh et al. used action research methods (analysis of documentation and observations) including qualitative and quantitative content analysis and thematic analysis to develop greater understanding about the strengths and challenges of community engagement in supporting ethical research practice [46]. Morin et al. used a rapid assessment model of data collection to examine the process of CAB development and the interaction between CAB members, research team members and research participants [22]. However, most of these authors have admitted the difficulty in measuring the success of any CE strategy or attributing the success of a research project to any particular CE strategy.

## Discussion

Community engagement is increasingly recognised as an important aspect of research involving populations. In the context of genomic research, the literature suggests that community engagement can assist in identifying and clarifying community members’ misunderstanding about the aims and intentions of the research project, as well as concerns about the procedures used to obtain data and samples [11]. It can also provide an opportunity for the research team to take into account the opinions of both the staff and community relating to issues pertinent to the study, allowing for adapting and modifying of information, messages and research methods [18,19,46].

This review also suggests that community engagement can enhance community members’ understanding of research and support the informed consent process, possibly by allowing information to be shared over time [10,44-49]. This is particularly important for genomic studies which involve complex scientific terminologies. Community engagement strategies such as consultations and interviews can also help identify community concerns and priorities related to the research study [11,19].

Various approaches to community engagement exist that can be explored. These include consultations with community leaders, community meetings, CAB, and focus group discussions. The choice of any particular approach will be determined by the goal of the engagement. However, as some authors have suggested, some form of ‘social mapping’ can provide valuable guidance for researchers to identify what strategies would work best in a given research context [5,50,51]. Tedrow et al. have recommended that it is important to create a tailored and yet flexible strategy to meet the uniqueness of each targeted community [39]. Marsh et al. have also suggested that community engagement strategies should be flexible to change as the research project and community engagement needs develop [16]. Also, it is important to use CE mechanisms that are familiar to the local community to limit any social disruption associated with the research [5].

There are also challenges with community engagement which need to be anticipated and addressed. These challenges vary depending on the nature of the research and the level and stages of the engagement effort. Given the lack of uniformity in the definitions for community and community engagement, predetermining an engagement strategy for a given research project can be a daunting task. Marsh et al. have suggested that community engagement focuses on issues directly experienced by the community and may not raise issues on less visible risks unless they are deliberately raised for discussion and consideration [18]. Community engagement is also complex and as Seeley et al. have reported, there is the need for *cultural sensitivity and ‘cultural intimacy’* [34]*.*

This review suggests that there is no ‘one-size-fits-all’ strategy to community engagement. The strategy to be adopted will depend on the nature of the research and the goals of the engagement. This may include providing information on various aspects of the project, consulting community leaders and representatives, actively involving or collaborating with various constituents within the community. Interestingly, most of the articles we reviewed included several strategies such as community meetings, focus groups and CABs within one project. Information giving and consultation appear to be two of the most important elements of community engagement in research in general and equally important in genomic studies. However, since active engagement often ends when samples have been obtained from research participants, it is still not clear if and when community engagement would be appropriate or desirable beyond the sample collection stage. Many of the CE strategies identified in this review are relevant for continuing to engage communities beyond sample collection. For example, working with CAB, organising community meetings or focus group discussions with patient groups provide an important opportunity to not only feedback findings but to discuss the progress of the project and also gauge community’s views on what future studies would be acceptable or indeed desirable [11]. Whether this is feasible, and what the impact would be on the research project and its outcomes, remain to be seen.

The CAB model presents an important opportunity to build trust and improve relationships between the research team and the community, as well as facilitate the recruitment and retention of study participants [22,52]. It could also be helpful in keeping the target community engaged for future uses of research samples through constant consultations and feedback between the CAB and research team. As Morin et al. have also suggested, the CAB can be a model through which the benefits of research can be made known and widely accepted within the larger community [22]. However, there are limitations to the CAB model that do not make it appropriate for use in all contexts.

Despite the limited evidence of what counts as effective CE in this review, we support calls for some form of social mapping exercise to enable research team members to identify what CE strategy would be appropriate in a given context [5,50,51]. Such an exercise will involve identifying local authority structures and existing channels of communication within the community. Research teams could also consider taking the following steps to evaluate their CE strategies. One, it is important for the goals of CE for any given research project to be clearly defined; two, some form of documentation of the CE process should be done including how the target community(ies) was identified, the strategies used, how these were modified in the course of the project, the challenges and lessons learnt and three; a formal evaluation should be conducted to assess if the CE goals were achieved, what strategies worked and what counts as good practice.

Research Ethics Committees could also play a valuable role in monitoring researcher-led community engagement activities, particularly in communities that are unfamiliar with genomic research, and where information giving and sharing, directed by the research team remains a primary focus of community engagement.

## Conclusion

Despite the lack of clear evidence from this review on what counts as effective community engagement and how the success of an engagement method or approach should be measured, we can draw a number of lessons from these examples to inform the development of CE strategies for genomic studies in Africa. Many of the strategies can support the early stages of a research project such as the recruitment of research participants. However, more research is needed to identify effective strategies that can be used to engage research participants and their communities beyond the sample collection stage. It would also be helpful to explore the views of local communities on issues such as broad consent and future unspecified use of samples as well as how these can be incorporated into the design of CE strategies and/or the development or review of guidelines for related genomics studies. The next phase of our research will take this a step further.

## Abbreviations

CE: Community engagement
CABs: Community advisory boards
CBPR: Community Based Participatory Research
IAP2: International Association of Public participation
